# Supplementary material for: Prevalence and associated factors of stunting among under-five children in Ethiopia: Application of marginal models analysis of 2016 Ethiopian demographic and health survey data
Source: PLoS One. 2023 Oct 31;18(10):e0293364. doi: 10.1371/journal.pone.0293364 (PMC10617690; doi:10.1371/journal.pone.0293364)
Supplement: S1 Checklist — (DOCX) [file pone.0293364.s001.docx]

**A STROBE statement checklist for study entitled *“Prevalence and correlates of stunting among under-five children in Ethiopia: Marginal Models Analysis of 2016 Ethiopian Demographic and Health Survey data”.***

| **Section** | | **Item No** | | | | **Recommendation** | | **Remark** | **Page No** |
| --- | --- | --- | --- | --- | --- | --- | --- | --- | --- |
| **Title and**  **abstract** | | 1 | | | | (a). Indicate the study’s design with a commonly used term in the title or the abstract | | **Done** | **1** |
|  |  |  |  |  |  | (b). Provide in the abstract an informative and balanced summary of what was done and what was found | | **Done** | **1&2** |
| **Introduction** | | | | | | | |  |  |
| Background/  rationale | | 2 | | | | Explain the scientific background and rationale for the investigation being reported | | **Done** | **3&4** |
| Objectives | | 3 | | | | State specific objectives, including any prespecified hypotheses | | **Done** | **4** |
| **Methods** | | | | | | | |  |  |
| Study design | | | 4 | | | Present key elements of study design early in the paper | | **Done** | **4** |
| Setting | | | 5 | | | Describe the setting, locations, and relevant dates, including periods of recruitment, exposure, follow-up, and data collection | | **Done** | **4&5** |
| Participants | | | 6 | | | (a). cohort study---Give the eligibility criteria, and the sources and methods of selection of participants. Describe methods of follow-up. | | **N/A** | **-** |
|  |  |  |  |  |  | Case-control study—Give the eligibility criteria, and the sources and methods of case ascertainment and control selection. Give the rationale for the choice of cases and controls | | **N/A** | **-** |
|  |  |  |  |  |  | Cross-sectional study—Give the eligibility criteria, and the sources and methods of selection of participants | | **Done** | **4** |
|  |  |  |  |  |  | (b) Cohort study—For matched studies, give matching criteria and number of exposed and unexposed | | **N/A** | **-** |
|  |  |  |  |  |  | Case-control study—For matched studies, give matching criteria and the number  of controls per case | | **N/A** | **-** |
| Variables | | | 7 | | | Clearly define all outcomes, exposures, predictors, potential confounders, and effect modifiers. Give diagnostic criteria, if applicable | | **Done** | **4&5** |
| Data sources/ measurement | | | 8* | | | For each variable of interest, give sources of data and details of methods of assessment (measurement). Describe comparability of assessment methods if there is more than one group | | **Done** | **4** |
| Bias | | | 9 | | | Describe any efforts to address potential sources of bias | | **Done** | **5** |
| Study size | | | 10 | | | Explain how the study size was arrived at | | **Done** | **4** |
| Quantitative variables | | | 11 | | | Explain how quantitative variables were handled in the analyses. If applicable, describe which groupings were chosen and why | | **Done** | **5** |
| Statistical methods | | | 12 | | | (a). Describe all statistical methods, including those used to control for confounding | | **Done** | **5-7** |
|  |  |  |  |  |  | (b). Describe any methods used to examine subgroups and interactions | | **N/A** | **-** |
|  |  |  |  |  |  | (c). Explain how missing data were addressed | | **Done** | **4** |
|  |  |  |  |  |  | d) Cohort study—If applicable, explain how loss to follow-up was addressed | | **N/A** | **-** |
|  |  |  |  |  |  | Case-control study—If applicable, explain how matching of cases and controls was addressed | | **N/A** | **-** |
|  |  |  |  |  |  | Cross-sectional study—If applicable, describe analytical methods taking account of sampling strategy | | **N/A** | **-** |
|  |  |  |  |  |  | (e). Describe any sensitivity analyses | | **N/A** | **-** |
| **Results** | | | | | | | |  |  |
| Participants | 13* | | | | (a). Report numbers of individuals at each stage of study—eg numbers potentially eligible, examined for eligibility, confirmed eligible, included in the study, completing follow-up, and analysed | | | **Done** | **8-18** |
|  |  |  |  |  | (b). Give reasons for non-participation at each stage | | | **N/A** | **-** |
|  |  |  |  |  | (c). Consider use of a flow diagram | | | **N/A** | **-** |
| Descriptive data | 14* | | | | (a). Give characteristics of study participants (eg demographic, clinical, social) and information on exposures and potential confounders | | | **Done** | **8&9** |
|  |  |  |  |  | (b). Indicate number of participants with missing data for each variable of interest | | | **NA** | **-** |
|  |  |  |  |  | (c) Cohort study—Summarise follow-up time (eg, average and total amount) | | | **N/A** | **-** |
| Outcome data | 15* | | | | Cohort study—Report numbers of outcome events or summary measures over time | | | **N/A** | **-** |
|  |  |  |  |  | Case-control study—Report numbers in each exposure category, or summary measures of exposure | | | **N/A** | **-** |
|  |  |  |  |  | Cross-sectional study—Report numbers of outcome events or summary measures | | | **Done** | **8&9** |
| Main results | 16 | | | | (a). Give unadjusted estimates and, if applicable, confounder-adjusted estimates and their precision (eg, 95% confidence interval). Make clear which confounders were adjusted for and why they were included | | | **Done** | **9-18** |
|  |  |  |  |  | (b). Report category boundaries when continuous variables were categorized | | | **Done** | **8, 14&18** |
|  |  |  |  |  | (c). If relevant, consider translating estimates of relative risk into absolute risk for a meaningful time period | | | **N/A** | **-** |
| Other analyses | 17 | | | | Report other analyses done—eg analyses of subgroups and interactions, and sensitivity analyses | | | **N/A** | **-** |
| **Discussion** | | | | | | | |  |  |
| Key results | | | | 18 | | | Summarise key results with reference to study objectives | **Done** | **19-22** |
| Limitations | | | | 19 | | | Discuss limitations of the study, taking into account sources of potential bias or imprecision. Discuss both direction and magnitude of any potential bias | **Done** | **22** |
| Interpretation | | | | 20 | | | Give a cautious overall interpretation of results considering objectives, limitations, multiplicity of analyses, results from similar studies, and other relevant evidence | **Done** | **22&23** |
| Generalizability | | | | 21 | | | Discuss the generalizability (external validity) of the study results | **Done** | **22&23** |
| **Other information** | | | | | | | |  |  |
| Funding | | | | 22 | | | Give the source of funding and the role of the funders for the present study and, the original study on which the present article is based | **N/A** | **-** |
