# Supplementary material for: Prevalence and associated factors of stunting among under-five children in Ethiopia: Application of marginal models analysis of 2016 Ethiopian demographic and health survey data
Source: PLoS One. 2023 Oct 31;18(10):e0293364. doi: 10.1371/journal.pone.0293364 (PMC10617690; doi:10.1371/journal.pone.0293364)
Supplement: Attachment — Submitted filename: Comments to the Author.docx [file pone.0293364.s003.docx]

**Comments to the Author**

**Overall**

The manuscript presents an important issue, to examine Prevalence and correlates of stunting among under-five children in Ethiopia: The author used 2016 EDHS data to examine the status and correlates of stunting in Ethiopia and used Marginal
models to analysis the data. The 2016 EDHS data source and title that the authors used for this study were outdated, and several scholars have conducted studies on the same title from various perspectives and using various models. However, the authors need to adequately describe the details in some sections. Please look into the following comments in detail.

**Abstract**

**1.** Under the conclusion section of the abstract (line 33), the author suggested that to mitigate/reduce childhood malnutrition better to work on improving the knowledge and practice of parents on appropriate young child feeding practices, frequent growth monitoring, and appropriate and timely interventions. This conclusion/suggestion is inconsistent with the findings of the study. The authors may reconsider this point in line with the objective and findings of the study.

Introduction

2. The introduction section is mainly focused on malnutrition rather than stunting, the author may add a paragraph about stunting.

## **Data Source**

3. Why did the author prefer to use the outdated 2016 EDHS data when the recent 2019 EDHS data is available?

**Statistical analysis**

4. The author may clearly specify why and when the two marginal models were used in this study.

Discussion and Conclusion

5. The paper focuses a lot on the different models, but not on the outcome of these tools. If the purpose is to compare models, then perhaps more comparisons of the different models should be presented.
