# Supplementary material for: Prevalence and associated factors of stunting among under-five children in Ethiopia: Application of marginal models analysis of 2016 Ethiopian demographic and health survey data
Source: PLoS One. 2023 Oct 31;18(10):e0293364. doi: 10.1371/journal.pone.0293364 (PMC10617690; doi:10.1371/journal.pone.0293364)
Supplement: Attachment — Submitted filename: Response to Reviewers .docx [file pone.0293364.s004.docx]

**Response to reviewers**

**Manuscript ID:** PONE-D-23-15241

**Research Title:** Prevalence and correlates of stunting among under-five children in Ethiopia:  Marginal models analysis of 2016 Ethiopian demographic and health survey data

**To:** Editor

**Re:** Response to reviewers

Dear Editor,

Thank you for allowing resubmission of our manuscript, with an opportunity to address the reviewers’ comments.

We, authors, would also like to express our gratitude to the anonymous reviewers for carefully reviewing the manuscript and for many thoughtful comments, which can enhance the readability and quality of this manuscript.

We are uploading (i) our point-by-point response to the comments (below) (response to reviewers), and (ii) an updated manuscript with changes being highlighted by yellow colour.

Best regards,

Wubishet Gezimu, corresponding author of the manuscript

***Reviewer #1:***

***Dear reviewer, thank you for reviewing and forwarding such important comments that enhance the readability and the quality of our manuscript.*** ***For easy check-up, our responses and all the changes in the manuscript are highlighted by blue colour.***

1. Under the conclusion section of the abstract (line 33), the author suggested that to mitigate/reduce childhood malnutrition better to work on improving the knowledge and practice of parents on appropriate young child feeding practices, frequent growth monitoring, and appropriate and timely interventions. This conclusion/suggestion is inconsistent with the findings of the study. The authors may reconsider this point in line with the objective and findings of the study.

**Authors’ Response:** We made correction.

2. The introduction section is mainly focused on malnutrition rather than stunting, the author may add a paragraph about stunting.

**Authors’ Response:** We have added a paragraph about stunting.

3. Why did the author prefer to use the outdated 2016 EDHS data when the recent 2019 EDHS data is available?
   **Authors’ Response:** We preferred to use 2016 EDHS data because the recent Mini 2019 EDHS data has many missing observations on some variables.
4. The author may clearly specify why and when the two marginal models were used in this study.
   **Authors’ Response:** Marginal models such as generalized estimating equations (GEE) and alternating logistic regression model (ALR) can be used for the data which has clustering nature. The main goal of the marginal models is to analyze the population-averaged effects of the given predictors on the binary response variable of interest.
5. The paper focuses a lot on the different models, but not on the outcome of these tools. If the purpose is to compare models, then perhaps more comparisons of the different models should be presented.

**Authors’ Response:** The study has compared the two models (GEE and ALR) and selected the better model to analyse data of childhood stunting. The outcomes/ results were based on the chosen model. On model comparison, we have used quasi-likelihood information criteria (QIC). The model which has the smaller QIC is chosen as the better model to analyse data. Thus, the study focuses both on models and outcome of the tools.

***Reviewer #2:***

***Dear reviewer, we are so much grateful for your very crucial and constructive comments that help to improve the quality of the manuscript. Your comments are so logical and very important. For easy check-up, our responses and all the changes in the manuscript are highlighted by blue colour.***

1. In the Title: Why do you use "correlates" rather than association?

**Authors’ Response:** We have corrected it. We have said, “Prevalence and associated factors of stunting among under-five children in Ethiopia:  Application of Marginal models using 2016 EDHS data”.

2. In the Abstract: Write the prevalence of stunting in the result section of the Abstract.
   **Authors’ Response:** We made correction. We have added prevalence of stunting in the result section of the abstract.
3. In the Introduction:
   ¬ Please write more about stunting than malnutrition since your title focuses on stunting.

**Authors’ Response:** We have added a paragraph about stunting in the introduction part of the manuscript.
¬ Please correct the following grammatical errors:
¬ On page 3, lines 50 and 51, "cause for almost half" by "of, and "communities of developing countries" by "in." And some others (check others).

**Authors’ Response:** We have corrected grammatical errors which were on page 3, line 50 and 51.
¬ The authors stated a regional variation of stunning/malnutrition in Ethiopia on page 3. Still, the author didn't include the region type as a single variable or not use a multilevel logistic model to examine regional variation as other previous studies found these stunting regional variations (as an example, you can look your reference # 19 conducted by W. Mekonnen Haileselassie et al., 2019).

**Authors’ Response:** We made correction on regional variation of stunting. It’s just to state the prevalence of stunting in the regions.
¬ And, if you look at this interesting article, I don't know the importance of this study (your study). You may need to apply the Generalized Estimating Equations and alternating logistic regression models even if it is not recommended to use them for this DHS dataset (read the Note on the methods and materials section)!

**Authors’ Response:** Marginal models such as generalized estimating equations (GEE) and alternating logistic regression model (ALR) can be used for the data which has clustering nature. The main goal of the marginal models is to analyze the population-averaged effects of the given predictors on the binary response variable of interest. EDHS data is a clustered data because for the purpose of administration in Ethiopia, regions are divided into zones, zones into weredas and weredas into lowest administrative unit or kebele. During the 2007 census, each kebele was sub-divided into census enumeration areas (EAs) or clusters which were convenient for the implementation of the census. Thus, the 2007 census conducted by the Central Statistical Agency (CSA) provided sampling frame from which the 2016 EDHS sample was drawn. Thus, it’s possible to use marginal models such as Generalized Estimating Equations (GEE) and Alternating Logistic Regressions (ALR) for EDHS data. Many articles are published using marginal models for DHS data.
¬ The authors have not stated clearly the gaps (ways of sampling design and clustering) with many previous related studies in Ethiopia. So please state the holes clearly in this section.

**Authors’ Response:** We have stated the gaps (ways of sampling design and clustering) related with previous studies in Ethiopia.

4. In the Methods and Materials:
   ¬ Please write the sampling design and clustering methods clearly since they are the gaps with other previous studies, as you said on page 4.

**Authors’ Response:** Sampling design and clustering methods are included in the methods and materials.
¬ Why did you use 626 clusters since there are 643 clusters in the 2016 EDHS data?

**Authors’ Response:** We used 626 clusters since there are 643 clusters in the 2016 EDHS data because the remaining 17 clusters had missing values on height-for-age (stunting) records. The complete height-for-age record was collected from 626 clusters.

¬ I don't know why you applied the Generalized Estimating Equations and alternating logistic regression models for this single DHS data. The data is neither Longitudinal (repeated) nor correlated/clustered data!

**Authors’ Response:** EDHS data is a clustered data because in Ethiopia for the purpose of administration, regions are divided into zones, zones into weredas and weredas into lowest administrative unit or kebele. During the 2007 census, each kebele was sub-divided into census enumeration areas (EAs) or clusters which were convenient for the implementation of the census. Thus, the 2007 census conducted by the Central Statistical Agency (CSA) provided sampling frame from which the 2016 EDHS sample was drawn. Thus, it’s possible to use marginal models such as Generalized Estimating Equations (GEE) and Alternating Logistic Regressions (ALR).
¬ Note! (1) The Generalized Estimating Equations (GEE) procedure extends the generalized linear model to allow for analysis of repeated measurements or other correlated observations, such as clustered data! (2) The GEE method was developed by Liang and Zeger (1986) to produce regression estimates when analyzing repeated measures with non-normal response variables. The response variable (Y) can be either categorical or continuous. (3) The alternating logistic regressions (ALR) algorithm of Carey, Zeger, and Diggle (1993) models the association between pairs of responses by using log odds ratios instead of using correlations, as ordinary GEEs do.
¬ So, what are the pairs of responses in this study dataset or 2016 EDHS datasets?

**Authors’ Response:** ALR measures the association between pairs of responses using log odds ratio i.e. ALR measures the association between two responses/ outcomes using log odds ratio. Assume $Y_{jk}$ and $Y_{jl}$ are two responses/ pair of responses where $Y_{jk}$represent child *k* from cluster *j* and $Y_{jl}$ represent child *l* from cluster *j*. Then, the ALR model measures the association of the two outcomes by

$logit P(Y_{jk}=1/Y_{jl}=y_{jl})=\gamma_{jkl}y_{jl}$+$\log\left( \frac{\pi_{\mathrm{jk}}{- v}_{\mathrm{jkl}}}{{1-\pi}_{\mathrm{jk}}-\pi_{\mathrm{jl}}+v_{\mathrm{jkl}}} \right)$

Where $\gamma_{jkl}$ be the log odds ratio between outcomes $Y_{jk} \& Y_{jl}$and $\pi_{jk}=P(Y_{jk}=1)$ and $v_{jkl}=P\left( Y_{jk}=1,Y_{jl}=1 \right).$

5. In the Results: Please correct the following grammatical errors:
   ¬ On lines 146 and 148, remove "who were" and "of" in "children who were included" and "male and of female".

**Authors’ Response:** We have corrected the grammatical errors which were existed on line 146 and 148.

6. In the conclusion: it needs more editions! So please use the edited conclusion as follows "GEE and ALR have been compared to analyze the average effect of predictors on the response variable. It is concluded that the ALR model with a measure of association exhibited a better fit for this data than the GEE models. This study suggests that a mother's education level, BMI, previous birth interval, and wealth index adversely affect childhood stunting. In contrast, the child's age and place of residence positively affect childhood stunting. However, in this study, the child's sex, mother's age at first birth, number of household members, number of antenatal care visits, birth order, source of drinking water, diarrhea recently and fever before two weeks of the survey were not significantly associated with childhood stunting. More importantly, this study contributes to understanding the individual and collective effect of maternal, socio-economic and child-related risk factors of childhood stunting in Ethiopia".

**Authors’ Response:** We have edited the conclusion based on your guidance.
7. In the Discussions: it also needs more grammatical editions!

**Authors’ Response:** We have edited/ corrected the grammatical errors which existed in the discussions.
¬ Finally, please write the limitations of the study!

**Authors’ Response**: We have added the limitations of the study in the manuscript.

Journal Requirements:

When submitting your revision, we need you to address these additional requirements.

1. Please ensure that your manuscript meets PLOS ONE's style requirements, including those for file naming. The PLOS ONE style templates can be found at https://journals.plos.org/plosone/s/file?id=wjVg/PLOSOne_formatting_sample_main_body.pdf and

<https://journals.plos.org/plosone/s/file?id=ba62/PLOSOne_formatting_sample_title_authors_affiliations.pdf>

**Authors’ Response**: Thank you so much for your suggestion. We ensure that our manuscript meets the all PLOS ONE's style requirements

2. We noticed you have some minor occurrence of overlapping text with the following previous publication(s), which needs to be addressed:

https://globalizationandhealth.biomedcentral.com/articles/10.1186/s12992-019-0505-7

https://journals.plos.org/plosone/article?id=10.1371%2Fjournal.pone.0256722

https://repository.ju.edu.et/bitstream/handle/123456789/3470/dani%20thesis%20%20to%20be%20printed.pdf?isAllowed=y&sequence=1

In your revision ensure you cite all your sources (including your own works), and quote or rephrase any duplicated text outside the methods section. Further consideration is dependent on these concerns being addressed.

**Authors’ Response**: Thanks a lot for this very important suggestion. We have your suggestion and accordingly made a significant changes (rephrased the duplicated texts) to our manuscript. Thanks once again.

3. In your Data Availability statement, you have not specified where the minimal data set underlying the results described in your manuscript can be found. PLOS defines a study's minimal data set as the underlying data used to reach the conclusions drawn in the manuscript and any additional data required to replicate the reported study findings in their entirety. All PLOS journals require that the minimal data set be made fully available. For more information about our data policy, please see http://journals.plos.org/plosone/s/data-availability.

Upon re-submitting your revised manuscript, please upload your study’s minimal underlying data set as either Supporting Information files or to a stable, public repository and include the relevant URLs, DOIs, or accession numbers within your revised cover letter. For a list of acceptable repositories, please see http://journals.plos.org/plosone/s/data-availability#loc-recommended-repositories. Any potentially identifying patient information must be fully anonymized.

Important: If there are ethical or legal restrictions to sharing your data publicly, please explain these restrictions in detail. Please see our guidelines for more information on what we consider unacceptable restrictions to publicly sharing data: http://journals.plos.org/plosone/s/data-availability#loc-unacceptable-data-access-restrictions. Note that it is not acceptable for the authors to be the sole named individuals responsible for ensuring data access.

We will update your Data Availability statement to reflect the information you provide in your cover letter.

**Authors’ Response**: Thanks so much. We have included the minimal underlying data set in the supporting information of the updated manuscript. Again we have uploaded it.

4. We note you have included a table to which you do not refer in the text of your manuscript. Please ensure that you refer to Table 5 in your text; if accepted, production will need this reference to link the reader to the Table.

**Authors’ Response**: Thanks a lot. We have corrected (referred table five in text) the mentioned section.
