# Supplementary material for: Prevalence and associated factors of stunting among under-five children in Ethiopia: Application of marginal models analysis of 2016 Ethiopian demographic and health survey data
Source: PLoS One. 2023 Oct 31;18(10):e0293364. doi: 10.1371/journal.pone.0293364 (PMC10617690; doi:10.1371/journal.pone.0293364)
Supplement: Attachment — Submitted filename: Comment for author-2.docx [file pone.0293364.s005.docx]

The authors responded most of my comments. One more comment for author is that please compare your results in discussion section with that of the previous study done by Wake et.al., (2023) <https://doi.org/10.1186/s13690-023-01090-7>
