# Supplementary material for: Prevalence and associated factors of stunting among under-five children in Ethiopia: Application of marginal models analysis of 2016 Ethiopian demographic and health survey data
Source: PLoS One. 2023 Oct 31;18(10):e0293364. doi: 10.1371/journal.pone.0293364 (PMC10617690; doi:10.1371/journal.pone.0293364)
Supplement: Attachment — Submitted filename: Response to Reviewers.docx [file pone.0293364.s006.docx]

Dear editor and reviewers, thanks a lot for the time with our manuscript so far. All your constructive comments and suggestions boosted up our manuscript’s scientific quality and readability. We have responded to Reviewer #1’s suggestions as mentioned bellow:

Reviewer #1:

The authors responded most of my comments. One more comment for author is that please compare your results in discussion section with that of the previous study done by Wake et.al., (2023)

<https://doi.org/10.1186/s13690-023-01090-7>

**Authors’ response:** Thank you so much for the suggestion. We have discussed our findings with the recommended literature and cited it accordingly as highlighted under the discussion section of the file. Thanks once again.

Journal Requirements:

Please review your reference list to ensure that it is complete and correct. If you have cited papers that have been retracted, please include the rationale for doing so in the manuscript text, or remove these references and replace them with relevant current references. Any changes to the reference list should be mentioned in the rebuttal letter that accompanies your revised manuscript. If you need to cite a retracted article, indicate the article’s retracted status in the References list and also include a citation and full reference for the retraction notice

**Authors’ response:** We have checked and corrected the references.
